# An Ultrasonic Rheometer to Measure Gas Absorption in Ionic Liquids: Design, Calibration and Testing

**DOI:** 10.3390/s20123544

**Published:** 2020-06-23

**Authors:** Michele Schirru, Michael Adler

**Affiliations:** AC2T Research GmbH, Viktor Kaplan-Straße 2/c, 2700 Wiener Neustadt, Austria; michael.adler@ac2t.at

**Keywords:** ultrasonic sensor, ionic liquid, viscosity measurement, reflection coefficient

## Abstract

The first goal of this study is to identify the ideal piezoelectric material for the manufacturing of rheological reflectance ultrasonic sensors. The second goal is to integrate the ultrasonic rheometer within a gas absorption reactor and to measure viscosity changes in an ionic liquid (IL) caused by gas absorption. To achieve the objectives, bismuth titanate, lead titanate, lead metaniobate and lead zirconate titanate materials in layer, tungsten bronze and perovskite structures were assembled on aluminum delay lines and tested under thermal cycling between room temperature and 150 °C. The results showed that lead metaniobate in tungsten bronze structure is the most suitable material for long time duration thermal cycling. Therefore, the ultrasonic rheometer was assembled using this material and installed in a pressurized reactor to test a reference IL at the operating conditions of 50 °C and at a pressure of 80 bar. The reference IL was saturated with nitrogen as well as hydrogen gas. Viscosity signals remained constant under the hydrogen atmosphere, while in nitrogen atmosphere the absorption of the gas lead to a rise in the value of viscosity.

## 1. Introduction

This study proposes an innovative ultrasonic reflectance rheological sensor for the testing of ionic liquids (IL). Ionic liquids are organic salts with melting points below 100 °C. These liquids have recently gained popularity in the industry because their lubrication properties are ideal for certain machinery and mechanical components [1]. The main advantages are very low volatility, high thermal stability and low flammability. All the same, this promising class of lubricant is still not fully characterized, and it is therefore important to study their rheology under operating conditions. ILs can be designed to have low gas absorption, though the extent of their gas absorption and potential preferential behavior towards some gas species is still a matter of research. The absorption of gas may change the viscosity of the ILs as gas molecules interact with the liquid and fill up free spaces [2], therefore the development of viscometric tests for ILs is of importance.

Viscosity is the key selection criteria for most machine applications. Therefore, the reliability during operation conditions and over time is of utmost importance. Furthermore, the online measurement of viscosity serves as a reliable tool in condition monitoring, as it is linked to the condition and chemistry of the lubricant. Mechanical rheometers are the standard instruments for the characterization of lubricating oils [3]. The rheological study of ILs by means of conventional steady shear or vibrational cantilever viscometers is made complex by the acidic or corrosive nature of some of these liquids towards metals, including steel, aluminum, gold and copper [4]. Another difficulty is that conventional rheometers cannot always be used in-line, for example in reactors, due to their size or operating specifications. Therefore, long-term tests are complicated with conventional instruments.

In this research work, an ultrasonic high frequency reflection rheometer is designed to avoid the presence of moving parts in the fluid and to perform such long-term testing in a pressurized reactor.

The ultrasonic reflectance method has a long history. Mason developed the first resonant measurement of fluid viscosity in the early 1950’s [5]. Lamb used resonant quartz at high frequency to measure viscosity at the operating shear rate of journal bearings [6]. This technique later evolved in the quartz microbalance and in methods to use resonant ultrasonic crystal to measure fluid mass, chemical composition and viscosity simultaneously [7,8]. A major drawback of this early method is the fragility of the resonating element when it is exposed to harsh environments. In the case of studying compounds such as ILs the electrodes of the microbalance would be quickly corroded by the salt solution. Recently, the use of a delay line enabled separating the piezoelectric element from harsh environmental condition. A delay line is a material interposed between the piezoelectric element and the fluid. This allows the study of flows that are not conventionally accessible with mechanical or resonant quartz rheometers. When a piezoelectric device uses a delay line, it is also referred to as a reflectance device. In a reflectance ultrasonic device, the ultrasonic wave propagates through the delay line and it is partially reflected at the delay line–fluid interface. The reflected energy holds information of the fluid structure. The ultrasonic reflectance methodology has been used for measurement of lubricant film thickness [9,10,11], chemical relaxation, absorption [12,13] and viscosity [14,15,16,17,18,19]. The measurement method is obvious and several algorithm and procedures for measurement have been tested. However, literature lacks information on the protocol to determine the best piezoelectric element for ultrasonic reflectance sensors.

The most common piezoelectric material used in these devices is lead–zirconate–titanate, most known as PZT. This material has gained popularity due to the high piezoelectric coefficient d33, an index of the amplitude response of the material and its low cost. In sensing applications, it is the recommended material for medical applications, hydrophones, nondestructive-testing immersion and contact transducers [20,21,22]. A long delay line is needed when considering this material for applications above 100 °C. Although different class of PZT can withstand temperature up to 300 °C, they are susceptible to high amplitude hysteresis if subjected to thermal cycling. This causes great complications in the sensor calibration over long-term operations.

An alternative to PZT is proposed in this research work. In particular, the performances of a reference PZT in perovskite structure is compared to a selection of bismuth titanate (BT), lead metaniobate (LM) and lead titanate (PbT) in various structures. This is needed because researchers compared the piezoelectric material substrate performances under thermal cycling [23], but the performances of reflectance devices were not evaluated in rheological operating conditions.

There are various reasons that justify the failure in the accurate selection of piezoelectric materials for ultrasonic reflectance devices. First, the material properties of the piezoelectric element are rarely considered in numerical approaches to correlate ultrasonic reflection to test sample properties. Rather the piezoelectric element is only the mean to produce the excitation and receive the ultrasonic wave reflected from the contact. Detailed numerical model and matrix approaches exist that consider the piezoelectric mechanical properties, but normally such models are simplified to obtain an easy-to-use solution of the reflectance problem [24].

Second, the design of an ultrasonic reflectance sensor would require knowledge of various disciplines. Mechanical engineering is needed to design the overall packaging and solving problems inherent to the integration of the sensor within the system under test conditions, to design appropriate bonding line and for the selection of packaging material (e.g., in case of passivation requirements). Basis of electrical engineering or mechatronics are needed to select appropriate electromagnetic noise rejection strategies, circuitry, and electrical load to interface with control instrumentation. Basics of material science are needed to fully understand the piezoelectric active element behavior and correctly interpret the response of the material under operating conditions. Finally, knowledge of acoustics is required to design the delay line to avoid unwanted reflections and to correctly interpret the sensor response. According to the state of the art and to the knowledge of the authors, only a few researchers worldwide have mastered all these disciplines in relation to the construction of reflectance sensor [25].

Therefore, it is not a surprise that some aspects of the construction of these sensors have been disregarded. Among these, the choice of piezo materials in relation to specific applications and the choice of the sensor calibration method are of special concern in this work.

In this work, a newly developed ultrasonic rheometer was employed in the test of the effect of gas absorption in ionic liquids. It is supposed that certain gases could be dispersed in the liquid mixture at high pressure and change the viscosity [26]. Gases dissolved in ILs under high pressure and temperature form a second phase, and it was shown that several liters of gas can be absorbed for each liter of lubricant [27]. However, the real effect of such gas absorption on the rheological properties of ionic liquids is unknown. Measuring and understanding this change is crucial to avoid catastrophic breakdown of machine due to lubrication failure. Gas absorption can be studied in reactors that introduce gas at high pressure in the ILs. The use of ultrasound technology enables the measurement of viscosity in real time at a high sampling rate that allows also to focus on transient phenomena.

To summarize, this study has the objective to select the optimum piezoelectric transducer material for long term duration rheological testing, design an ultrasonic rheometer employing such material, and to use this sensor for the in situ measurement of gas absorption in pressurized ionic liquids.

## 2. Materials and Methods

### 2.1. Background on Reflection Coefficient Measurement Techniques

**Figure 1:**
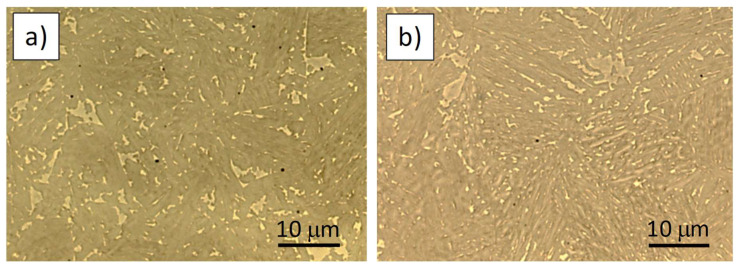
(**a**) Schematic representation of ultrasonic reflection in an ultrasonic viscometer; (**b**) reflection and transmission at the matching layer interface.

Figure 1 schematically shows the operating principle of an ultrasonic reflectance sensor.

A piezoelectric transducer is bonded on a substrate called delay line. The function of the delay line is to separate the piezoelectric transducer from the fluid surface to protect the fragile piezoelectric material from harsh environment, while providing a suitable acoustic path length. The piezoelectric transducer is the active element of the sensor and generates the ultrasonic shear wave. In a reflectance sensor, this ultrasonic wave propagates through the delay line until it reaches the fluid interface. At the liquid boundary, a part of the energy is reflected and a part is transmitted. The total amount of energy reflected is a function of the properties of the materials at the interface and measured as reflection coefficient. The reflection coefficient in its simplest form can be measured as [28]:

(1) $$R=\frac{Z_{dl}-Z_{f}}{Z_{dl}+Z_{f}}$$

where R is the reflection coefficient, $Z_{dl}$ is the acoustic impedance of the delay line and $Z_{f}$ is the acoustic impedance of the fluid. The reflection coefficient is very close to 1 when the impedance of the delay line is much higher than the one of the fluids. This means that approximately 100% of the ultrasonic energy is reflected and no information of the liquid substrate is measurable. This phenomenon is called acoustic mismatch [29] and quarter wavelength front faces are used to enhance the measurement sensitivity, as shown in Figure 1b. The matching layer is chosen to have a λ/4 thickness and an acoustic impedance that is equal to [30]:

(2) $$Z_{m}=\sqrt{Z_{dl}Z_{f}}$$

where $Z_{m}$ is the acoustic impedance of the matching layer. When the matching layer is added to the system, the total reflection coefficient for the three-layered system that includes the delay line, the matching layer and the fluid is [31]:

(3) $$R=\sqrt{\frac{Z_{m}^{4}+2Z_{f}^{2}Z_{dl}^{2}-2\left|Z_{f}\right|Z_{dl}Z_{m}^{2}}{Z_{m}^{4}+2Z_{f}^{2}Z_{dl}^{2}+2\left|Z_{f}\right|Z_{dl}Z_{m}^{2}}}$$

Figure 1b schematically shows the physical reason that leads to this increment in sensitivity. The reflections inside the matching layer *R_ml_* are in destructive interference with the ultrasonic waves incident (*I*) to the matching layer. In case of perfect interference, this interaction would lead to zero reflected energy. The amount of energy reflected from the polymer layer depends on the viscosity at the oil interface, this is a function of the impedance of the liquid. The relation correlating the impedance of the liquid *Z_f_* with viscosity is [16]:

(4) $$Z_{f}=\sqrt{i\omega \eta \rho_{f}}$$

where $\rho_{f}$ is the liquid density and ω is the piezoelectric transducer rotational frequency. Therefore, a change in viscosity leads to a change in the amount of reflected energy from the matching layer. The reflection coefficient *R* is correlated directly to viscosity as:

(5) $$\eta =\frac{\left|Z_{f}^{2}\right|}{2\omega \rho_{f}}$$

It was noticed [32] that the viscosity reflection curves acquired using an ultrasonic rheometer are comparable with the theoretical plot of Equation (3). This was valid for both, Newtonian oils and complex non-Newtonian fluids. The correlation is exponential:

(6) $$\eta =a*e^{bR}$$

The coefficients *a* and *b* in Equation (3) are characteristic for each individual sensor and they are derived for this research work in the “Results” section. Equations (1)–(6) show that the measurement of viscosity using the reflection coefficient theory requires the knowledge of the liquid density and speed of sound in the liquid, where the speed of sound is related to the acoustic impedance *Z_f_*. A full characterization of the mechanical and acoustical properties of the IL during the test is complex because it would require testing the superimposed effects of temperature and pressure on density and speed of sound [33]. Further, the speed of sound of shear waves is a complex parameter with both a real and an imaginary component whose determination is complex and not subject to any standard [34], such relation is highlighted in Equation (5). To reduce measurement uncertainty, in this work, the reflection coefficient is converted in viscosity using the empirical correlation shown in Equation (6).

### 2.2. Background on Ultrasonic Viscometry

Viscometer measurements depend on the shear rate, pressure, and temperature at which the measurement is executed. Standard viscometers may apply the shear with a mechanical rotating body and operate with steady shear rate up to 10 kHz [3]. Ultrasonic reflectance viscometers operate at high ultrasonic frequencies (> 1 MHz) using shear vibrations to displace the liquid medium. The Cox Merz rule correlate steady shear measurement to the rotational frequency methods [35]:

(7) $$\eta_{\omega }=\eta_{\dot{\gamma }}$$

where $\eta_{\omega }$ is the viscosity measured at the rotational frequency ω and $\eta_{\dot{\gamma }}$ is the viscosity measured at the steady shear rate $\dot{\gamma }$. This equation states that the viscosity measured with an oscillatory or ultrasonic viscometer at a rotational frequency is equivalent to the viscosity measured at the correspondent steady shear rate. Equation (7) is proven valid for Newtonian and base oils, but it is not always correct for complex formulations [36]. Historically, this relation has been used to correlate the viscosity at zero shear to the measurement of rotational viscometers up to a few kHz [37]. Bair [36] showed that such relation is valid also at higher vibrational frequencies by using a Carreau relation to correlate the low steady shear viscosity to high rotational shear viscosity:

(8) $$\eta_{0}={\left(1+{\left(\tau_{rel}\omega \right)}^{2}\right)}^{\frac{\left(N-1\right)}{2}}$$

where $\eta_{0}$ is the low steady shear viscosity, $\tau_{rel}$ is the relaxation time and *N* is the Carreau coefficient. In complex oil formulations this relation is valid only for the base of the fluid because high frequency oscillations cannot displace high molecular weight particles [38] thus the viscosity measured is equivalent to the viscosity measured at infinite shear rate *η_∞_* [36]. This was proven [31,39] by tests conducted on solutions of squalane and polyisoprene (SQL+PIP). It was noticed that the measurement of the SQL base was comparable with what expected from Equations (7) and (8), while the PIP additive was not measured at high shear.

Equation (8) was also applied in the study of ionic liquids [40]. The shear viscosity at high frequency was simulated using molecular dynamics and values for the coefficient *N* were calculated at different temperatures and pressures. Literature provides the data for the relaxation time of the ionic liquid base tested in this work [41]. In the material section, the existing molecular dynamics and chemical characterization of ionic liquids it is therefore used to estimate the expected value of viscosity for the IL under examination.

### 2.3. Materials

#### 2.3.1. Piezoelectric Materials

Table 1 shows the characteristics of the piezoelectric transducers tested in this research work. All the piezoelectric substrates were chosen to operate at the frequency of 5 MHz. The choice of the candidate materials was made based on three parameters [42]: Curie temperature, the piezoelectric constant d33 and the dielectric thermal constant. The first parameter determines the temperature limit at which depolarization of the piezoelectric element occurs. For most industrial applications, this temperature should be at least 200 °C, although some special applications for example in the oil & gas or nuclear sectors require operation temperatures above 500 °C. The d33 is an indicator of the amplitude of the vibrational energy generated by the piezoelectric element in the direction parallel to the plane of polarization. The higher the d33 the higher the amplitude as well as the signal to noise ratio (SNR) is expected for the selected piezoelectric material. The d33 is regarded as one of the main indicators for piezoelectric performance. Finally, the thermal constant indicates how the amplitude of the reflected signal changes with a change in temperature. In this work only elements with a thermal constant between 1 and 4 °C^−1^ are considered to achieve a low change in amplitude response when subject to a large temperature gradient.

Further, the piezoelectric materials for this study were selected in a mix of the main three structures to assess the influence of the piezoelectric structure on the response. These are: perovskite, tungsten bronze and layered.

**Figure 2:**
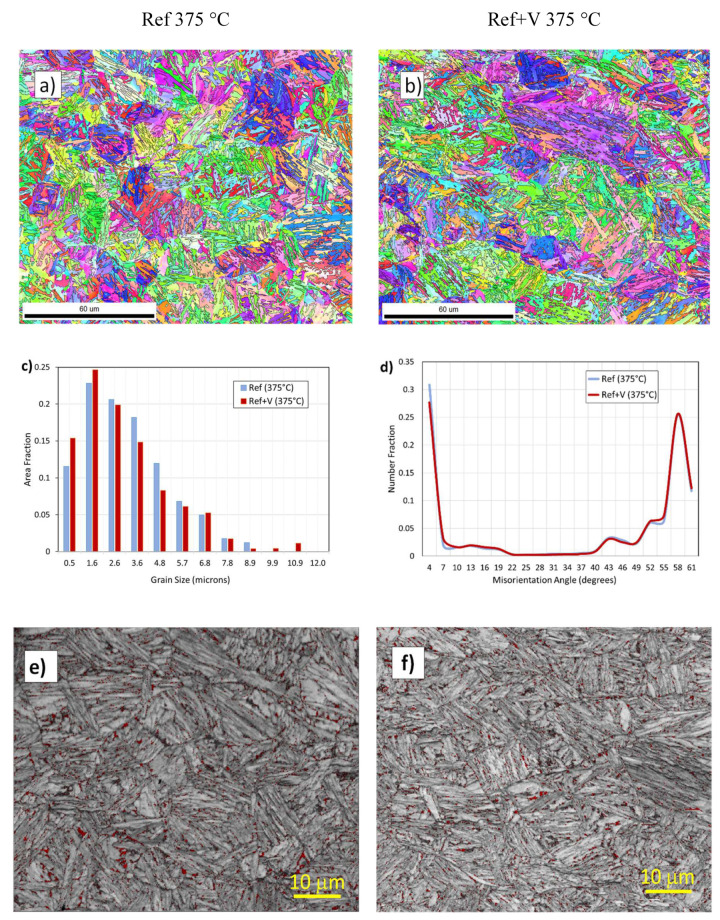
Comparison between piezoelectric and thermal constants for different class of piezoelectric materials.

Figure 2 shows a comparative graph between the response amplitude of different piezoelectric elements at varying temperatures. This graph helps visualizing how a high amplitude response is accompanied by a high thermal dielectric constant that is normally associated with poor stability to thermal cycling. In the next section, these material structures amplitude responses are compared to select the best element for rheological applications. Lead–zirconate–titanate—commonly known as PZT—was chosen as a reference material because most of state-of-the-art sensing applications use this class of piezoelectric materials as active elements [43,44,45,46].

#### 2.3.2. Test Samples

The ionic liquid test sample was an imidazolium based ionic liquid formulated for low gas solubility. This means that the base was an ionic liquid, EMIM in this case that was mixed with an additive package. The physical characteristics of the reference ionic liquid were as reported in Table 2. The characteristics were evaluated using a Stabinger type viscometer [47].

It is highlighted that the viscosity measured in accordance with standard ASTM D341−17 [49] is executed at low steady shear. As discussed in the background section, the viscosity measured with the ultrasonic viscometer was equivalent to the base viscosity of EMIM because the polymer package could not be measured by high frequency ultrasonic waves.

Given the knowledge of the EMIM base viscosity, the EMIM relaxation time and Carreau coefficients [40,41,50] and using Equation (8), it was possible to estimate the expected range of viscosity at the operating temperature of 50 °C to be in the range 15 to 20 mPas.

Further, viscosity standard Newtonian oils were chosen to calibrate the ultrasonic sensor. The standard S60 and N14 mineral oil from Canon^®^ were chosen in this work. These were Newtonian mineral oils according to the standards ASTM D445/D446 and ISO 3104/3105 for conventional steady shear viscometers calibration. Further, research works had proven the validity of Newtonian mineral oils as mean for calibration of ultrasonic instruments [15]. The viscosity of the calibrating oils was chosen to include the expected viscosity limits of the test. Table 3 reports the viscosity of the calibrating oil in the temperatures range considered.

### 2.4. Testing Methodology and Data Postprocessing

#### Calibration and Measurement Methodology

**Figure 3:**
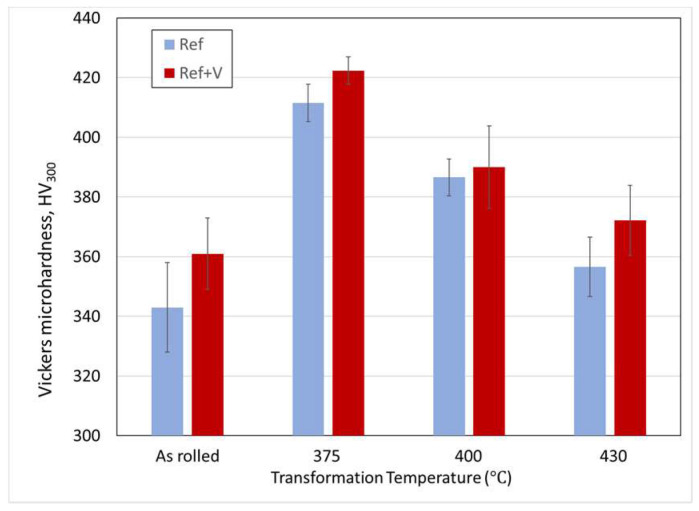
Ultrasonic sensor test and development flow chart.

Figure 3 shows the test methodology stages. These were: piezo material selection, ultrasonic rheometer calibration and test in the gas absorption reactor. The first stage consists of identifying the optimal piezoelectric materials for reliable rheological testing. This was done by testing a variety of piezoelectric materials and structures assembled in customized delay lines. The second stage consists of calibrating the sensor with the best piezoelectric material obtained from the tests in stage 1. Finally, only when these two stages were successful, the sensor was mounted in the gas absorption reactor for testing.

A clinical trial type approach was applied to design the experiments at each testing stage [51]. In particular, the following principles were followed in this study:

- Sample randomization.
- Test randomization.
- Blinding, for stage two only.
- Test repetitions.

**Stage 1: Piezoelectric material and data postprocessing**

**Figure 4:**
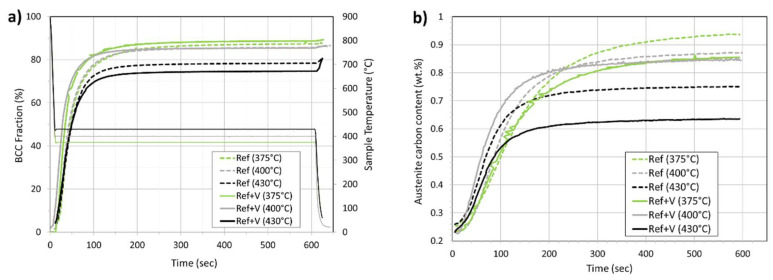
(**a**) Schematic representation of the ultrasonic reflectance sensor and test setup; (**b**) two ultrasonic sensors equipped with K-type thermocouples.

A total of 18 aluminum 6061 delay lines were manufactured and three sensors were constructed for each piezoelectric material, as reported in Table 1. Each piezoelectric transducer was bonded to the delay line with a high temperature strain gauge epoxy resin. This required that the piezoelectric material be loaded and cured in the oven to guarantee the optimal bonding line. A repeatable bonding line was obtained by using a jigging mechanism that allowed the piezoelectric element to be bonded and cured simultaneously for 10 delay line assemblies. This provided a repeatable manufacturing procedure for the test sensors. Once the piezoelectric element was bonded, the delay line was assembled in a simple packaging, as shown schematically in Figure 4a,b. The connection to a RF connector was obtained with a copper wire of 30 mm in length soldered between the connector and the top electrode of the piezoelectric transducer, while the aluminum casing was used as the ground.

**Figure 5:**
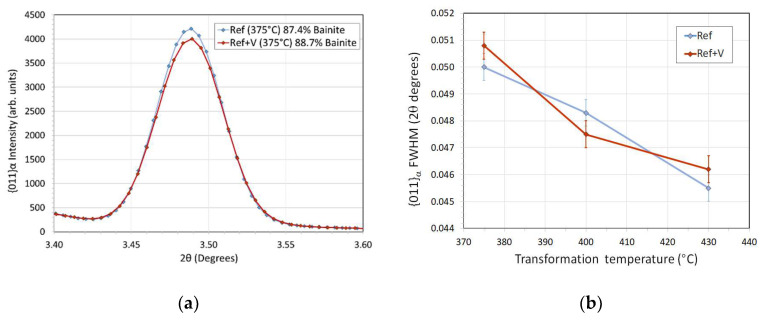
Schematic representation of the test sensor measurement chain.

**Figure 6:**
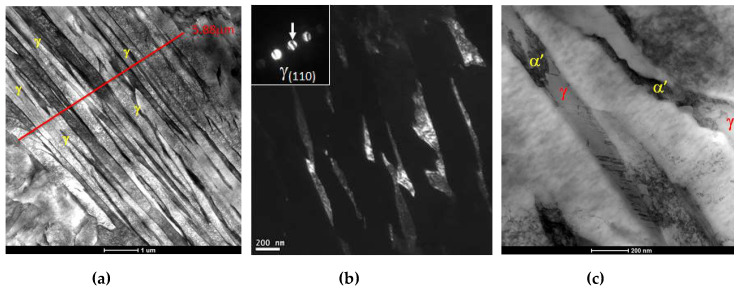
Schematic representation of the test thermal cycling.

Figure 5 schematically shows the measurement chain. A waveform generator was controlled by a PC software and used to generate a 5-MHz sine burst with a length of 10 cycles. The sensor response was filtered, displayed on an oscilloscope and postprocessed in the control PC. The time domain signals from the sensor were acquired throughout a thermal cycle. This is shown in Figure 6. The test sensors were slowly heat up to the maximum temperature of 150 °C and then cooled down to room temperature. This temperature was chosen to not exceed the maximum operating temperature of the bond line and thus to avoid the debonding of the piezoelectric element from the delay line. The temperature was held constant for 20 min to make sure that the temperature was constant across the piezoelectric element and delay line. Each thermal calibration was repeated 3 times for each sensor. No repetition was conducted at the same day to reduce the electrical heating and enhance statistical validity.

**Figure 7:**
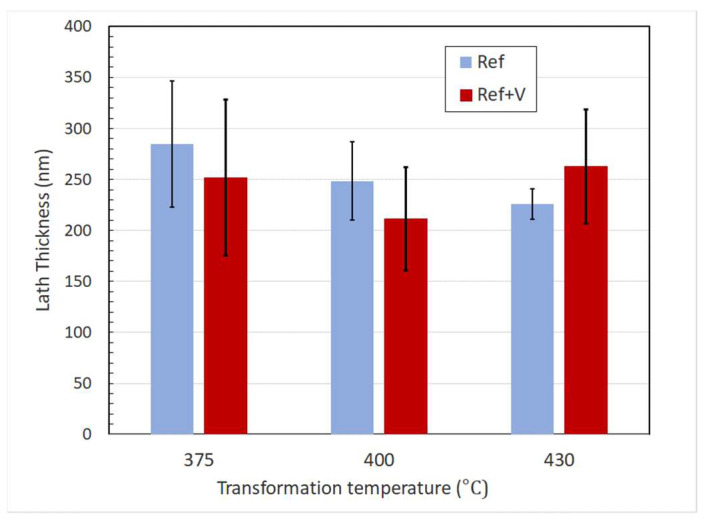
(**a**) Time domain ultrasonic signal, the reflection and measurement noise are highlighted; (**b**) FFT of ultrasonic signal.

The data postprocessing is as follows. The first-time domain ultrasonic reflection from the delay line was acquired at the end of each step of the thermal cycle. The time domain signal was then converted in the frequency domain using a Fast Fourier Transform (FFT) algorithm. Figure 7 shows an example of ultrasonic time domain signal, Figure 7a and the FFT amplitude of such signal, Figure 7b.

Consequently, a series of performance parameters were calculated for each experiment. These include the bandwidth (*BW*), the signal to noise ratio of the transducer [51], the linearity of the response and the data correlation to the linear response (R^2^). The bandwidth was calculated as:

(9) $$BW=\left(\frac{f_{u}-f_{l}}{f_{c}}\right)\times 100$$

Moreover, the *SNR* was calculated as:

(10) $$SNR=\left(\frac{A_{IR}-A_{N}}{A_{IR}}\right)\times 100$$

In Equation (9), *BW* is the bandwidth, *f_u_* the upper frequency limit at −6 dB from the maximum amplitude, *f_l_* is the lower frequency limit at −6 dB from the maximum amplitude and *f_c_* is the center frequency of the response. In Equation (10), *SNR* is the signal to noise ratio, *A_IR_* is the amplitude of the first reflection and *A_N_* is the amplitude of the noise.

**Stage 2: Calibration of the rheological sensor**

**Figure 8:**
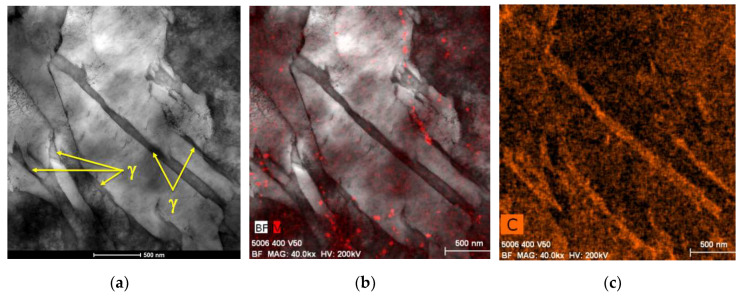
Schematic representation of the ultrasonic rheometer.

Figure 8 shows schematically the ultrasonic rheometer assembled with the selected piezoelectric element. The materials and bonding procedures are executed as in stage 1; however, the geometry includes an NPT 1/8 thread to allow mounting on the test reactor as shown in the next section. Further a front face of polymeric material 50-μm-thick is added as quarter wavelength acoustic lens to enhance the ultrasonic response sensitivity [52].

The sensor was calibrated to obtain the reflection coefficient–viscosity curves to use as look up tables in the gas absorption reactor test. These look up tables were obtained by measuring the response of the sensor to two viscosity standard Newtonian reference S60 and N14 oils by Canon^®^ over the temperature range 25 °C to 100 °C. The reflection coefficient *R(T_i_)* was measured experimentally as:

(11) $$R\left(T_{i}\right)=\frac{A_{m}\left(T_{i}\right)}{A_{R}\left(T_{i}\right)}$$

where *A_R_(T)* is the reference amplitude of the sensor response and *A_m_(T)* is the measurement response of the sensor in the presence of the test fluid. The reference amplitude *A_R_(T)* was measured by measuring the reflected amplitude from the delay line air interface at different temperature. An experimental setup as in Figure 4a was used to heat up the sensor and record the reflected amplitudes *A_R_(T)* across a range of temperatures. The measurement response *A_m_(T)* is obtained by adding a layer of Canon^®^ calibrated oil in the petri dish. This testing stage was executed by a colleague of the author with no prior knowledge on ultrasonic technology to introduce a form of blinding. Blinding is essential to eliminate the author bias. However, to maintain quality control on the tests, the author established the test and postprocessing protocol.

**Stage 3: Gas Absorption Reactor Testing and Postprocessing**

**Figure 9:**
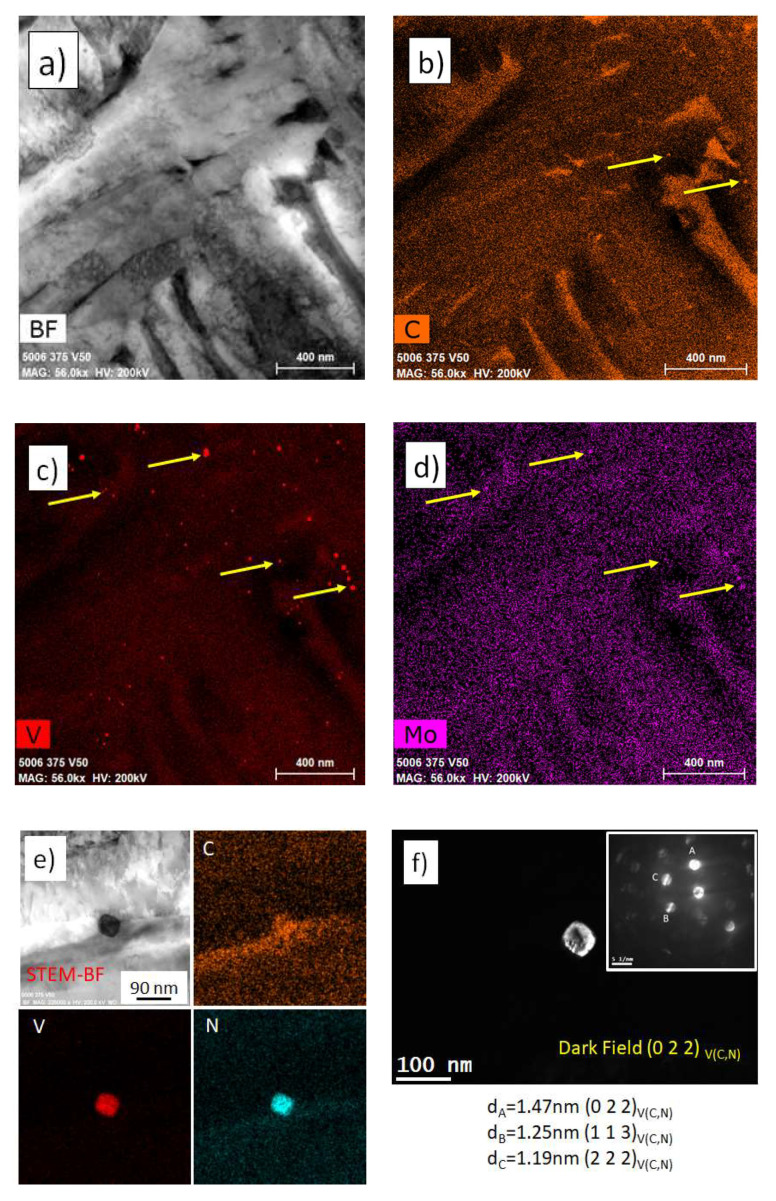
Schematic representation of the gas absorption test setup. The ultrasonic measurement chain is highlighted in bold lines.

Figure 9 showed the test setup in the pressure reactor. It was divided in two main blocks, the gas absorption test rig (thin line) and the ultrasonic sensor measurement chain (bold line). The gas (in this work nitrogen or hydrogen was used) was fed in the test rig and was pressurized in a pressure reactor produced by Parr Instruments^®^. The reactor was modified to include a high-pressure pipe to feed the gas into the test fluid reactor. This second reactor comprised also an agitator that allows the gradual mixing of the gas in the test fluid. Both reactors had thermal coils to control the temperature of both, the gas and the fluid. The pressure reactor, the valves, the agitator and the temperature systems were controlled by a control PC that was independent from the ultrasonic sensors control. Temperature and pressure data from both the liquid and the gas reactors were recorded at the rate of 0.2 Hz by the PLC software.

**Figure 10:**
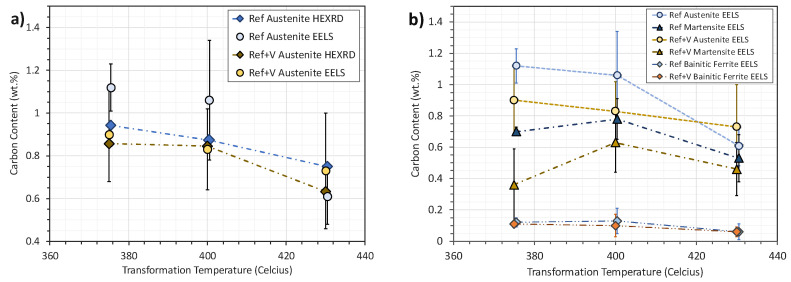
Ultrasonic sensor mounted in the gas absorption test rig.

Figure 10 shows that the ultrasonic sensor was mounted on the bottom of the lubricant reactor, and the matching layer of the sensor in Figure 8 was in direct contact with the IL. A PC software controls an arbitrary waveform generator to produce a 10 cycle 5 MHz sine wave burst electric signal to excite the ultrasonic sensor. The ultrasonic reflection from the sensor was visualized on an oscilloscope, recorded, and converted in a reflection coefficient value by the software. The obtained reflection coefficient was then used to calculate the viscosity. The PC also controlled the NI^®^9213 DAQmX thermocouple system that provided the temperature reference for the ultrasonic signals postprocessing. Both the ultrasonic and temperature measurements were performed with a 1-Hz sampling rate.

## 3. Results

**Stage 1: Piezoelectric Material Selection**

**Figure 11:**
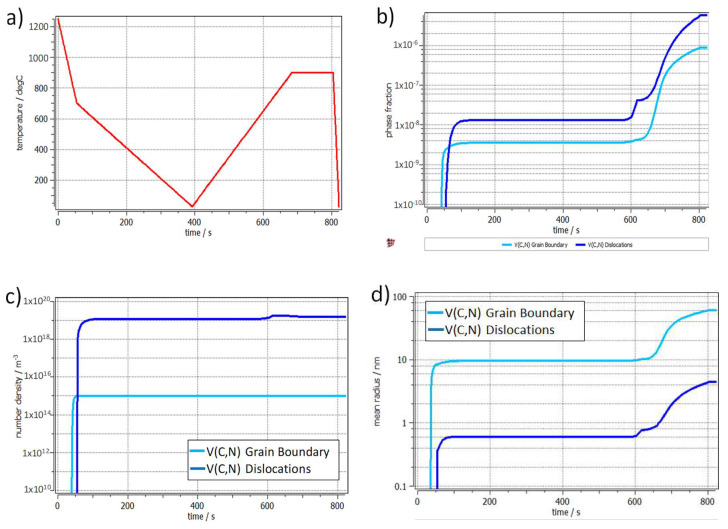
Test results of LM3 substrates.

**Figure 12:**
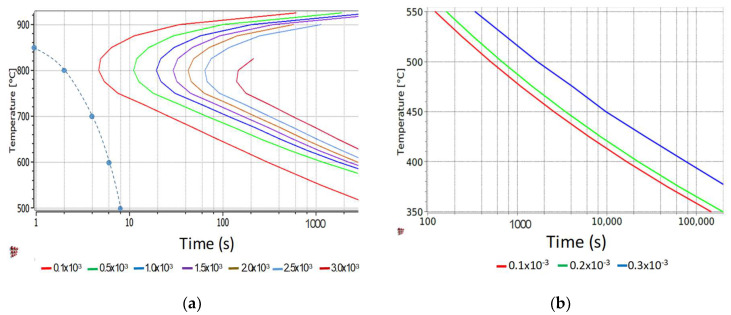
Test results from the PZT transducer.

Figure 11 and Figure 12 show the amplitude–temperature measurement obtained for different piezoelectric materials. These are representative results of the full test matrix. The LM samples show no thermal hysteresis and the relation between amplitude and temperature is linear. The outliers in Figure 11 were not random but were part of the first thermal cycle. It was noticed for more than 60% of the piezoelectric material testing that the first thermal cycle was characterized by a stabilization of the response of the piezoelectric element. It is shown in literature, in fact, that the first thermal cycling for a piezoelectric material could produce a stabilization response [23]. This testing proves that such thermal stabilization occurs also for ultrasonic reflectance devices. Further, these results highlight the importance of multiple thermal cycling to reference appropriately ultrasonic reflectance sensors. It was noticed in all the experiments (with the exception of the tests on PZT) that this behavior was not repeated starting from the second thermal cycling.

Figure 12 shows that the response of the PZT substrate was not constant over temperature and time. Each thermal cycling was characterized by a strong hysteresis and the amplitude response decreased with each test repetition. Therefore, it was not possible to derive performance indicators from the testing of the PZT sensors. This behavior was repeatable for all the sensors manufactured with this piezoelectric material. This PZT material was chosen as representative of the main grade used in industry and this grade was also characterized by an advantageous dielectric thermal coefficient. Nevertheless, the thermal response was poor and not ideal for reflectance measurements. The results for all the piezoelectric materials tested are reported in Table 4, and in the online data repository, see Supplementary Materials [53]. This table highlights the performance parameters that were chosen to evaluate the test ultrasonic sensors.

The samples PT, LM1 and LM3 show an increment in amplitude with temperature and this was extremely advantageous as most of the rheological applications in industry involve high operating temperature. The obvious advantage of this increment in reflected amplitude was a higher SNR at standard operating temperatures encountered in industrial applications. The *BW* was low for all sensors; however, this did not surprise, as the test sensors were constructed without backing material. Overall, the material LM3 showed the best performance response under thermal cycling and a very good correlation between various heating cycles. Table 4 highlights that some performance parameters for the PZT sample could not be calculated due to the lack of response stabilization, therefore, PZT could not be considered as a suitable candidate for long term ultrasonic reflectance applications in which thermal cycling was involved. BiT showed an almost constant amplitude with changes in temperature; however, the *SNR* was too low for the planned application. This type of material is suggested, however, for rheological tests to be conducted at temperature above 400 °C where high response stability is required and where no other commercially available material can operate due to the high Curie temperature required.

**Stage 2: Referencing Rheometer**

**Figure 13:**
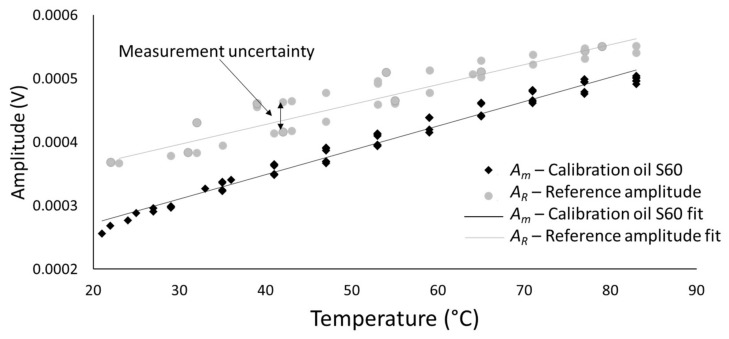
Example of referencing and measurement function for a measurement on the S60 sample.

**Figure 14:**
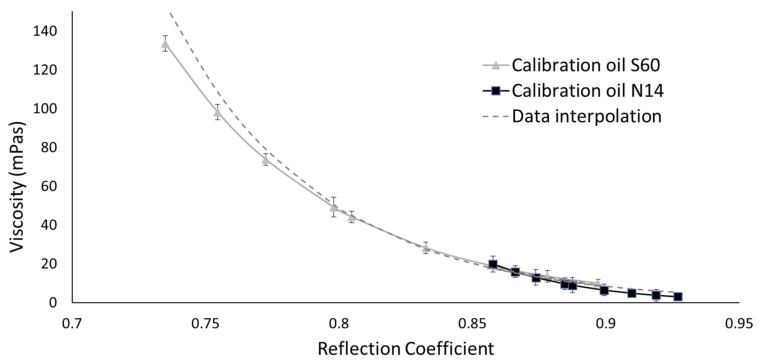
Reflection coefficient–viscosity diagram for the S60 and N14 calibration oils. A common exponential fitting is obtained for these two calibration oils over the range of temperature 20–100 °C.

The rheometer in Figure 8 was assembled using the material LM3, a lithium niobite in tungsten bronze structure. The thermal referencing was repeated using the same setup as for the tests in stage 1 to obtain the amplitude *A_R_* over a range of temperature. The test on the hot plate was then repeated using two calibration oils with different viscosity ranges, S60 and N14. This was done to confirm the reliability of the viscometer and to establish a correlation between measured reflection coefficient *R* and shear viscosity. Figure 13 shows the linear interpolation functions that were used to interpolate the reflection coefficient, while Figure 14 shows the reflection coefficient against temperature and the reflection coefficient against viscosity *η*.

The reflection coefficients were calculated using Equation (12) with the values of amplitude at different temperatures, as shown in Figure 13, in case of an air interface to obtain the reference amplitude A_R_, and in case of an oil interface to obtain the measurement amplitude *A_m_*. These results show that the measurement of the viscometer is highly repeatable, and that the reflection coefficient measurement is independent of temperature because the response of the S60 and N14 oils superimpose. The sensor calibration data and results are available in the online data repository [53]. Table 2 shows that this happened at different temperatures for the two reference oils. This allow to establish a simple correlation between reflection coefficient and viscosity, as outlined in the methodology section:

(12) $$\eta =e^{20.261-17.6R}$$

**Stage 3: Test in Gas Absorption Reactor**

**Figure 15:**
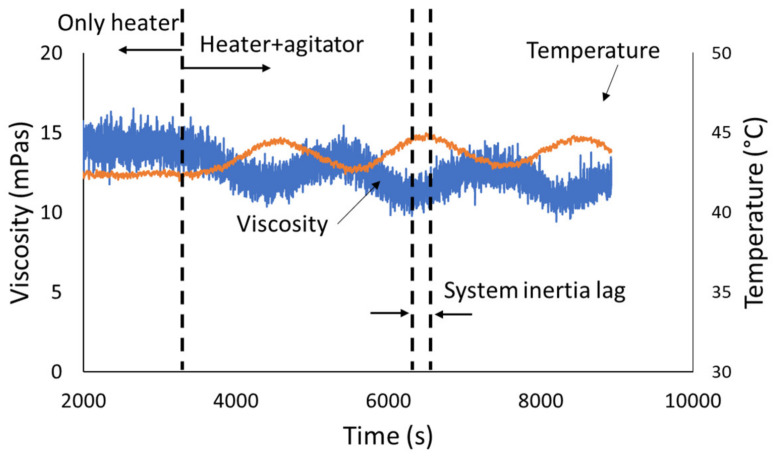
Ultrasonic rheometer response under thermal cycling. No noise is introduced by the agitator.

The sensor installed in the reactor was tested against electromagnetic noise that may be present due to the agitator or other external sources. Figure 15 shows that the response of the sensor to temperature changes in the reactor was very fast and that even the change in viscosity due to the temperature variations induced by the agitator was recorded. No noise source was recorded. This experiment was also conducted to establish the inertia of the sensor response with temperature variation within the reactors. This was estimated to be about 300 s.

**Figure 16:**
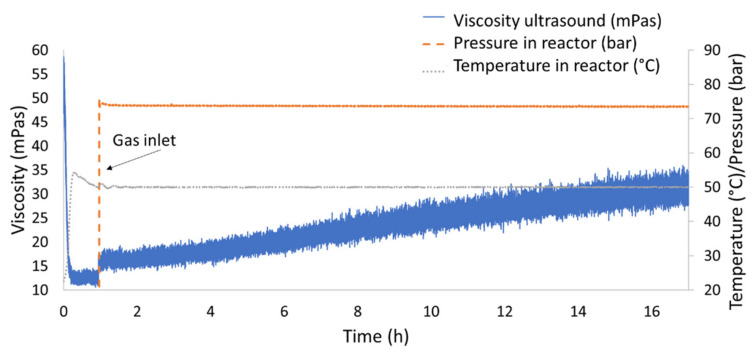
Viscosity change due to gas absorption of IL in a saturated nitrogen atmosphere.

**Figure 17:**
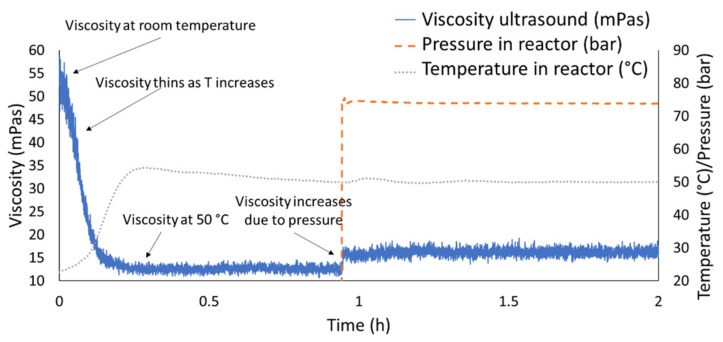
Detail of the first two hours of testing in case of nitrogen saturated atmosphere.

Figure 16 shows the results of the first test in which nitrogen was added to the IL. Figure 17 highlights the first hour of operation of this test. At first, the reactor was brought to the constant temperature of 50 °C before the pressure was applied and therefore the gas was mixed in the reactor containing the IL. Figure 17 highlights the sensitivity to temperature and pressure changes. In particular, it could be noticed how the viscosity drops when temperature rise, such decrement was in line with what expected in literature [31] and with what was outlined in the materials section Table 2. On the contrary an increment in pressure resulted in an exponential increment in viscosity [32].

**Figure 18:**
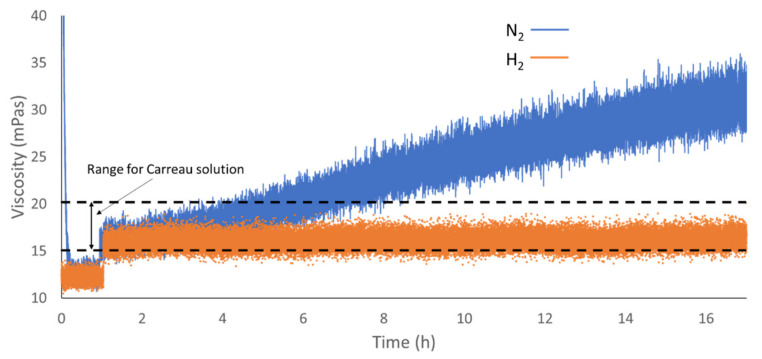
Viscosity change of the IL over time in case of nitrogen and hydrogen saturation.

Figure 18 compares the experiments conducted for both nitrogen and hydrogen. The experiment with hydrogen was conducted with the temperature set at 50 °C and so no decrement in viscosity was noticed at the beginning of the test. The Figure 18 also highlights the expected range for the Carreau solution as calculated in the “materials” section. The environmental conditions of the tests with hydrogen and nitrogen were the same. The tests show that the accuracy of the measurement was of ±3 mPas in average, but it was as high as ±5 mPas in the last hours of operations during the tests with nitrogen. The tests showed high repeatability and measurement overlap in case of repetitions in the same conditions within the reactor. Each test was conducted on separate days and along a two-week span. The comparison shows that hydrogen was not absorbed by the IL and there was no effect on the ultrasonic response and therefore no change in the measured viscosity. On the other hand, nitrogen was absorbed by the IL at the operating pressure [53]. Viscosity changes due to gas absorption were measured for hydrocarbon-based lubricants [54], however this behavior was not documented so far for ILs. This highlights the selective nature of the additive package to gas absorption. In fact, in case of the nitrogen experiments, the rate of absorption was such that after 17 h of operation the viscosity doubled. Further, the tests showed that saturation was not achieved even after 17 h of testing. Furthermore, Figure 18 shows that the viscosity measured with the ultrasonic sensor was in line with the expected viscosity for the EMIM base, as reported in Table 2, and did not take into consideration the high molecular weight additive package. The results confirm that a Carreau model [36,40] estimate the expected viscosity of the IL base at high rotational frequency. Further studies were needed to determine the exact Carreau exponents for EMIM based ionic liquid formulations to achieve a higher precision in the model prediction.

## 4. Conclusions

This study proposes a wholistic approach to develop and test ultrasonic reflectance rheometers. In particular, the active piezoelectric element was selected to be reliable over long period of operations and a wide variation of applications. As a first usability test, the ultrasonic sensor was applied to examine the effect of gas absorption on ILs. This is a class of lubricant that is difficult to study in situ with conventional steady shear or cantilever rheometer due to their corrosive nature against steady shear and cantilever rheometer plating materials, such as gold. This instrument opens the possibility to fully characterize the rheological characteristics of ILs in harsh environmental conditions. The main results of this study are the following:

1. Thermal cycling within the operating temperature range was required upon constructing reflectance sensor devices to allow the setting of piezoelectric material properties. At least three thermal cycles should be executed to verify the sensor response stability.
2. Lead metaniobate in tungsten bronze structure was the ideal piezoelectric material for the construction of ultrasonic reflectance rheometers. This selection was based on overall bandwidth, signal to noise ratio and response linearity in the range of temperature 20 °C to 150 °C. Overall, BiT showed the highest stability to temperature and it was the recommended material for special operations above 400 °C.
3. Conventional ultrasonic sensors employing PZT were characterized by reflected amplitudes that change over time with thermal cycling. This makes the referencing of such piezoelectric device impossible for long operation periods.
4. The viscosity–reflection coefficient calibration curves were not dependent on temperature. This was proven by the fact that the reflection coefficients for two calibrated oils with identical viscosity at different temperatures overlap in a viscosity–reflection coefficient diagram. This was of high importance to reduce the measurement uncertainty when testing a sample of unknown acoustic and mechanical properties and to simplify referencing procedures for ultrasonic devices.
5. The ultrasonic rheometer developed in this work was stable over long-time operations and the corrosive nature of the IL under study did not influence the sensor response. This proves that polymeric front faces were suitable for long term testing of ILs.
6. The measurement of the influence of gas absorption to the viscosity of ILs was made possible by implementing the sensor system into a gas absorption reactor. In particular, the reference IL did not show changes in viscosity when absorbing hydrogen, while the absorption of nitrogen at the same conditions led to significant changes in the viscosity measured. This showed the importance to execute gas absorption tests on lubricants as mechanism operating pressures may induce changes in viscosity that were not predicted by conventional rheological testing.

## Figures and Tables

**Table 1:** Test piezoelectric materials and structures.

Material	Structure	Curie T (°C)	d33 (10^–12^ C/N)	Dielectric Constant, Thermal Stability (°C^−1^)
BiT	Layer	650	21	1.0
PT	Perovskite	380	72	1.8
LM1	Tung. Bronze	480	90	1.6
LM2	Tung. Bronze	320	185	1.8
LM3	Tung. Bronze	280	62	2.2
Hard PZT	Perovskite	330	245	4.0

**Table 2:** Physical properties of the test ionic liquid (IL).

Ionic Liquid	
Property	Value
Viscosity @ 40 °C (mPas)—100 Hz	50.0
Viscosity EMIM base @ 40 °C (mPas)—100 Hz [48]	20.0
Viscosity @ 100 °C (mPas)—100 Hz	8.5
VI	159.4
Density @ 40 °C (kg/m^3^)	1242.6
Density @ 100 °C (kg/m^3^)	1204.2
Relaxation time EMIM (ns)	4.0
Carreau viscosity (based on [41,42,48]) @ 50 °C (mPas)	15.0 to 20.0

**Table 3:** Standard calibration oil viscosity–temperature data.

Viscosity Standard Oils		
Temperature (°C)	Viscosity S60 (mPas)	Viscosity N14 (mPas)
20	141.0	24.0
25	104.0	20.0
40	47.0	11.0
50	30.0	8.2
80	10.0	3.8
100	6.2	2.6

**Table 4:** Performance parameters for the test piezoelectric transducers.

Material	R^2^	Slope (V/°C)	Bandwidth (%)	SNR (%)
BiT	0.89 ± 0.09	−1.27 e^−6^ ± 3.67 e^−8^	21.0 ± 4.0	81 ± 15
PT	0.69 ± 0.24	1.37 e^−5^ ± 3.79 e^−6^	18.0 ± 2.4	82 ± 11
LM1	0.64 ± 0.28	2.00 e^−5^ ± 1.49 e^−5^	11.5 ± 0.5	94 ± 4
LM2	0.97 ± 0.02	−2.50 e^−3^ ± 3.64 e^−4^	12.5 ± 0.15	94 ± 3
LM3	0.98 ± 0.01	1.63 e^−3^ ± 2.70 e^−4^	12.5 ± 0.07	95 ± 2
Hard PZT	-	-	12.2 ± 0.15	95 ± 4

## Supplementary Materials

Data are available at https://triboportal.ac2t.at/. Please contact the authors of the paper to obtain the login credentials to the data repository.
